# Characteristics and Outcomes of Implementing Emergency Department-based Intensive Care Units: A Scoping Review

**DOI:** 10.5811/westjem.24874

**Published:** 2024-11-27

**Authors:** Jutamas Saoraya, Liran Shechtman, Paweenuch Bootjeamjai, Khrongwong Musikatavorn, Federico Angriman

**Affiliations:** * Chulalongkorn University, Faculty of Medicine, Division of Academic Affairs, Bangkok, Thailand; † Chulalongkorn University, Faculty of Medicine, Department of Emergency Medicine, Bangkok, Thailand; ‡ King Chulalongkorn Memorial Hospital, Thai Red Cross Society, Bangkok, Thailand; § University of Toronto, Interdepartmental Division of Critical Care Medicine, Toronto, Canada; ∥ Sunnybrook Health Sciences Centre, Department of Critical Care Medicine, Toronto, Canada; ¶ Chulalongkorn University, Department of Anesthesiology, Bangkok, Thailand; # Chulalongkorn University, Faculty of Medicine, Department of Medicine, Bangkok, Thailand

## Abstract

**Introduction:**

The prolonged stay of critically ill patients in the emergency department (ED) may lead to worse clinical outcomes. An emergency department (ED)-based intensive care unit (ICU) is one of the proposed solutions to deliver critical care in the ED. We thus aimed to characterize existent ED-ICU models and their reported association with clinical outcomes in critically ill adult patients.

**Methods:**

We searched the Ovid MEDLINE database from inception to October 2, 2023. We included studies that report an ED-ICU structure, defined as a space capable of providing ICU-level care within or adjacent to the ED, and its characteristics. We excluded personnel-focused intervention (without the presence of a separated space) or a space without ICU-level care capability. We collected information on process measures, patient-related outcomes, and cost-related outcomes.

**Results:**

We screened 2,824 studies, of which 125 full-text articles were assessed for eligibility and 31 studies were included in this scoping review. Studies reported on 14 ED-ICUs across seven countries, with capacities ranging from 3–17 beds. All ED-ICUs served early and ongoing critical care needs in the ED, including three distinct themes: short-stay; palliative care; and disaster-response ICUs. Implementing the ED-ICU was associated with decreased time to ICU-level care and reduced number of inpatient ICU admissions, but it was not consistently associated with improved survival.

**Conclusion:**

Several ED-ICUs have been established around the world with different characteristics depending on local needs. Implementation of the ED-ICU may be associated with improved clinical outcomes and patient flow.

## INTRODUCTION

Emergency department (ED) crowding is a recognized global health problem.^1^ Crowding can manifest as prolonged boarding of critically ill patients in the ED.^2^ In turn, delayed admission to the intensive care unit (ICU) may lead to clinically worse outcomes, including increased hospital stay and all-cause mortality.^3^ ^,^ ^4^ One strategy to mitigate this problem is to provide critical care in the ED. Several studies have identified different models of providing critical care in the ED, including geography-based and personnel-focused models.^5^ The personnel-focused model highlights the need to have early critical care consultation, while the geography-based model involves the expansion of ICU beds, either in the existing inpatient ICU or in the ED (the latter is generally called the ED-based ICU or the ED-ICU model).^5^ Theoretically, the ED-ICU model could provide timely critical care to patients in need; establishing it, therefore, represents a promising strategy to deal with ED crowding.^6^ ^–^ ^8^ Despite its promising nature, the specific characteristics of different ED-ICUs across the world have not been described. It is also uncertain whether the application of this ED-ICU model to an existing, operating ED affects its processes and clinical outcomes, in addition to costs.

In this scoping review we aimed to explore the characteristics (eg, capacity, staffing, and utilization) of implemented ED-ICU models. We further sought to explore how ED-ICUs affect process measures, in addition to clinically relevant outcomes and healthcare-associated costs.

## METHODS

### Data Sources

We searched the Ovid MEDLINE database from inception until October 2, 2023 using a search strategy developed with an experienced medical librarian (see Supplementary Table S1). We specifically chose to limit the Ovid MEDLINE search since we expected that this database would result in the highest yield of articles for the topic of interest. Keywords for the search strategy included emergency department, intensive care unit, and ED ICU. There were no language or study design restrictions. We screened the references of the included studies and manually searched them to identify additional eligible papers.

### Study Selection

We included all studies that reported an ED-ICU model structure, defined as a dedicated physical space with intensive care capacity within or adjacent to the ED, and its characteristics or outcomes. We captured information on reported characteristics such as number of ICU beds, staffing, reasons for ED-ICU initiation, and admission criteria. Information on reported outcomes including process measures, patient-related outcomes, and cost-related outcomes was also collected. We excluded studies that reported only on personnel-focused models of critical care delivery in the ED (ie, without a dedicated physical area), or a physical space that could not provide organ support measures that are core to an ICU-level care (eg, invasive mechanical ventilation or vasopressor support). Two authors independently screened abstracts and full texts (JS, LS, or PB). Conflicts were resolved by consensus through discussions held in a virtual meeting.

### Data Extraction and Synthesis

Specific data extracted from the included full texts included the name of the ED-ICU model, country, institution, year of establishment (and reason), number of beds, type of staffing, admission criteria, yearly census of the corresponding ED, utilization of ED-ICU, ED-ICU length of stay (LOS), ED-ICU reported mortality, description of process measures, patient-related or cost-related outcomes, first author and year of publication, study design, funding, and potential conflict of interests. Data was extracted independently into a pre-piloted data abstraction form by two authors (JS, LS, or PB). Conflicts were resolved by consensus through discussions held in a virtual meeting. We summarized the characteristics of each ICU using descriptive statistics as feasible, and we identified themes of utilization of the ED-ICU. The reported outcomes were grouped into three categories: 1) process measures; 2) patient-related clinical outcomes; and 3) cost-related considerations.

The protocol of this scoping review was registered and can be accessed on Open Science Framework (OSF) website (registration DOI: https://doi.org/10.17605/OSF.IO/MEVQB). This report follows the PRISMA extension for scoping reviews.^9^

## RESULTS

We screened 2,824 studies and assessed125 full-text articles for eligibility, of which 31 were included in the present scoping review (Figure 1). Of the 31 included studies, there was one prospective cohort study,^10^ 23 retrospective cohort studies,^6^ ^–^ ^8^ ^,^ ^11^ ^–^ ^30^ one systematic review,^31^ four narrative reviews,^2^ ^,^ ^5^ ^,^ ^32^ ^,^ ^33^ one report,^34^ and one commentary.^5^ These studies reported on 14 different ED-ICUs. Table 1 provides the characteristics of these 14 ED-ICUs. Supplementary Table S2 and S3 provide further details of ED-ICUs and each study.

**Figure.**
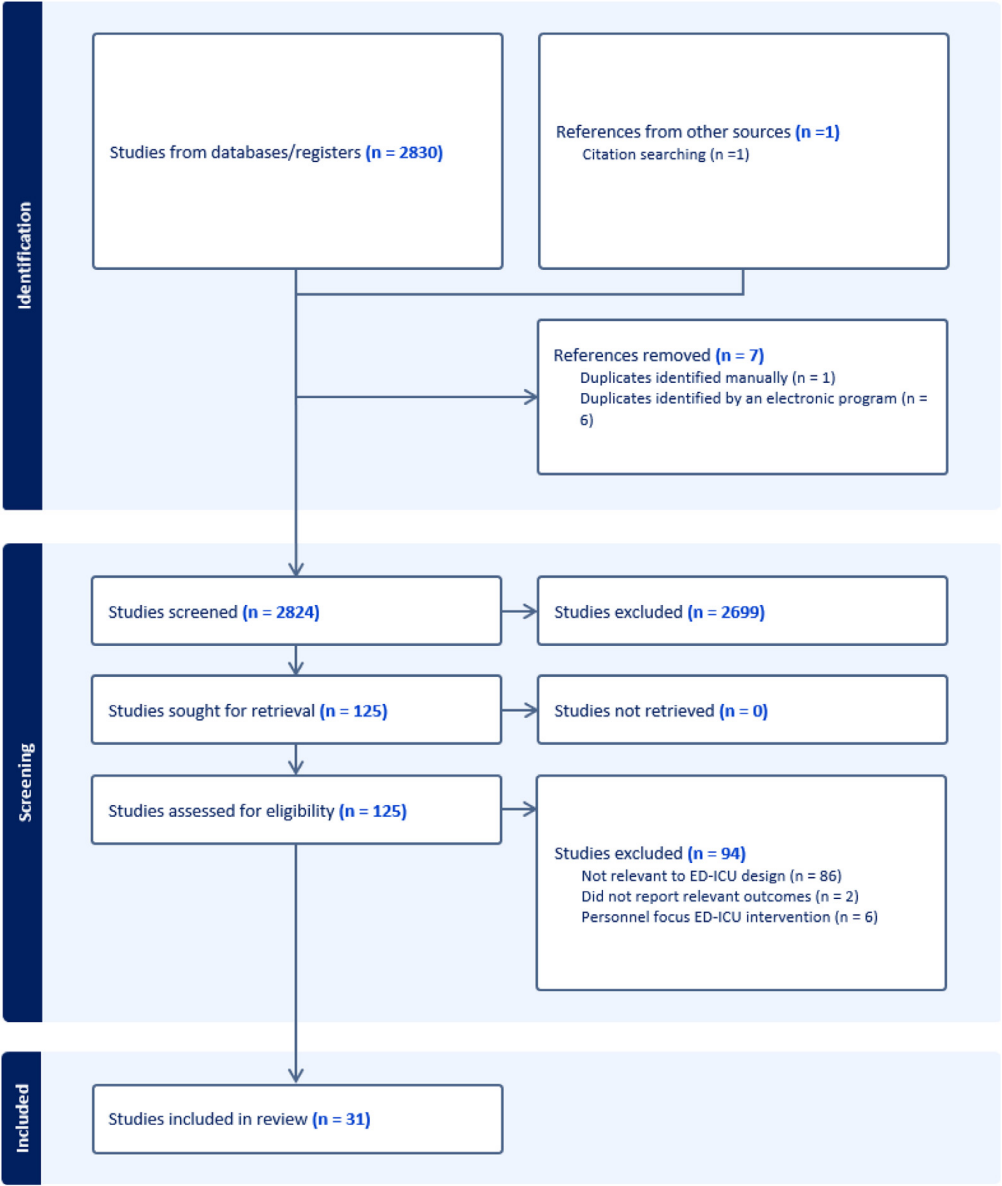
PRISMA chart for scoping review impact of intensive care units within emergency departments. *ED-ICU*, emergency department-based intensive care unit.

**Table.** Characteristics of emergency department-based intensive care units.

Name	Country	Institution	Reason for initiation	Admission criteria	Available beds
Emergency critical care center (EC3)^2^ ^,^ ^5^ ^,^ ^6^ ^,^ ^18^ ^–^ ^22^ ^,^ ^25^ ^–^ ^33^ ^,^ ^35^	United States	University of Michigan	ED boarding of ICU patients	Ongoing critical care need (even when other ICU bed is available)	9
Resuscitation and critical care unit (ResCCU)^2^ ^,^ ^5^ ^,^ ^8^ ^,^ ^17^ ^,^ ^23^ ^,^ ^32^	United States	Hospital of the University of Pennsylvania	ED boarding of ICU patients	Ongoing critical care need (even when other ICU bed is available)	6
Resuscitation and acute critical care unit (RACC)^2^ ^,^ ^5^ ^,^ ^32^ ^,^ ^33^	United States	Stony Brook University Medical Center	ED boarding of ICU patients	Ongoing critical care (when an ICU bed is not available)	3
ED intensive care unit (EDICU)^10^ ^,^ ^31^	Turkey	Hacettepe University	ED boarding of ICU patients	Ongoing critical care (when an ICU bed is not available)	8
ED ICU^5^ ^,^ ^14^	France	Amiens University Medical Center	To manage general critical care; eg, stroke patients	Not reported	6
ED intensive care unit (EDICU)^12^ ^,^ ^31^	Taiwan	Chang Gung Memorial Hospital	ED boarding of ICU patients	Ongoing critical care (when an ICU bed is not available)	14
Shock room^11^	Belgium	Erasme University Hospital	Facilitating rapid diagnosis and management of acutely ill patients	Unstable patients	4
ED-ICU^15^	Brazil	Instituto Central do Hospital das Clínicas da Faculdade de Medicina da Universidade de São Paulo	Not reported	Ongoing critical care (when an ICU bed is not available)	17
Emergency ICU (EICU)^7^	Republic of Korea	Seoul National University Hospital	ED boarding of ICU patients	Ongoing critical care need (even when other ICU bed is available)	12
Emergency ICU (EICU)^24^	Taiwan	Taipei Veterans General Hospital	ED boarding of ICU patients	Ongoing critical care (when an ICU bed is not available)	13
ED-ICU^16^	Taiwan	Taipei Medical University Hospital	Reservoir of critical care for hospital	Ongoing critical care	8
Emergency intensive care unit (EICU)^13^	United States	Bellevue Hospital Center	Disaster (hurricane)	Ongoing critical care before transfer	10
ED-ICU^34^	United States	Mount Sinai Hospital	Disaster (COVID-19)	Ongoing critical care need	13
Not reported^5^	United States	Henry Ford Hospital	ED boarding of ICU patients	Ongoing critical care (when an ICU bed is not available)	16

*ED*, emergency department; *EM*, emergency medicine; *EP*, emergency physician; *CCM*, critical care medicine; *COVID-19*, coronavirus disease 2019; *ICU*, intensive care unit.

### Main Characteristics of ED-ICUs

The ED-ICUs are described using different names or abbreviations, such as emergency department intensive care unit (with the abbreviations EDICU, ED ICU, or ED-ICU), emergency ICU (EICU), emergency critical care center (EC3), resuscitation and critical care unit (ResCCU), resuscitation and acute critical care unit (RACC), or shock room. The identified ED-ICUs were deployed in seven countries: six in the United States; three in Taiwan; and one each in Korea, France, Belgium, Turkey, and Brazil. While most ED-ICUs had one or two studies that reported on their overall structures, the EC3 in the US had 18 related articles.^2^ ^,^ ^5^ ^,^ ^6^ ^,^ ^18^ ^–^ ^22^ ^,^ ^25^ ^–^ ^33^ ^,^ ^35^ Bed capacity ranged from 3–17 beds with a median of 10 beds (interquartile range 7–13). Six ED-ICUs were reported to not be able to admit patients when inpatient ICUs were available, but three were reported to do so (see Table 1). Several common staffing patterns were observed in these ED-ICUs; for example, the presence of 1) emergency physicians, with or without training in critical care medicine, 2) registered nurses with a nurse-to-patient ratio of approximately 1:2, and 3) interdisciplinary team members, including respiratory therapists or pharmacists. One ED-ICU operated only on weekdays.^8^ ^,^ ^17^ ^,^ ^23^

### Overarching Themes

Three themes emerged from reviewing the unique utilization of these ED-ICUs: namely the ED-ICU as a short-stay, palliative care, or disaster-response ICU. First, ED-ICUs could provide short-stay intensive care for critically ill patients who were expected to recover quickly after a period of intensive observation or treatment (eg, patients with acute poisoning, upper gastrointestinal [GI] bleeding, diabetic ketoacidosis [DKA], or minor intracranial hemorrhage).^17^ ^,^ ^18^ ^,^ ^22^ ^,^ ^23^ ^,^ ^26^ Second, when inpatient ICU beds are full, patients who are unlikely to survive could be alternatively admitted to the ED-ICUs. Such ED-ICUs, thus serving as a palliative-care ICU, could be a place where physicians initiate end-of-life care discussions and palliative care consultations.^10^ ^,^ ^15^ ^,^ ^24^ Third, ED-ICUs were used for disaster response. For example, in the event of a hurricane or the coronavirus disease 2019 pandemic, temporary ED-ICUs were set up to increase hospital-bed capacity.^13^ ^,^ ^34^

### Process Measures

The ED-ICUs have been shown to potentially improve several process measures, including decreased time from ED presentation to deployment of ICU-level care.^6^ ^,^ ^7^ ^,^ ^17^ ^,^ ^18^ ^,^ ^26^ A retrospective cohort of 349,310 ED visits revealed that critically ill patients receive ICU-level care with a median of 1.9 hours earlier after opening an ED-ICU compared to before opening.^6^ Critically ill patients admitted to an ED-ICU had 27.5% shorter boarding time than patients admitted to other ICUs.^7^ Implementing the ED-ICU model resulted in an increase in the number of patients who received evidence-based critical care in the ED.^14^ ^,^ ^27^ For example, in patients presenting with acute ischemic stroke, the presence of an ED-ICU was associated with an increase in thrombolysis rate.^14^ Furthermore, among intubated patients in the ED, establishing an ED-ICU increased the number of patients receiving a lung protective ventilation strategy at the time of ED departure (65.8% vs 43.1%).^27^

Moreover, an ED-ICU model could facilitate the reduction of inpatient ICU admission and preserve inpatient ICU capacity. Opening an ED-ICU was associated with a reduction in the odds of overall admission to an inpatient ICU (adjusted odds ratio [aOR] 0.80; 95% confidence interval [CI], 0.76–0.83).^6^ Furthermore, reduced inpatient ICU admissions were found in cohorts of patients with DKA or upper GI bleeding after the ED-ICU implementation.^18^ ^,^ ^26^ In another setting, a study showed that the opening of an ED-ICU was associated with a decrease in ICU admission in the first month after opening among adult patients with sepsis.^8^

### Patient-Related Outcomes

Overall, the reported LOS of patients in the ED-ICUs varied widely. The ED-ICUs that admitted patients with acute poisoning, DKA, or minor intracranial hemorrhage reported their ED-ICU LOS as follows: 72% of patients with acute poisoning had an LOS of less than 24 hours^23^; the mean LOS for DKA was 18.1 hours^18^; and the median LOS for minor intracranial hemorrhage was 15.7 hours.^22^ In contrast, the other two ED-ICUs that admitted patients with a worse overall prognosis reported a median ED-LOS of 120 hours (interquartile range [IQR] 4 hours-49 days),^10^ and a median of 3.2 days (IQR 1.9–5.9 days).^15^ Similarly, a wide range of mortality rates were also observed, from less than 1% to ≈75%. Dispositions of patients in ED-ICUs varied; they could be subsequently transferred to inpatient ICUs, transferred to general wards, or discharged home.

We found conflicting data on the association of an ED-ICU model implementation with patient-related outcomes. For example, some studies showed that an ED-ICU was associated with improved clinical outcomes.^6^ ^,^ ^14^ ^,^ ^25^ In an adjusted analysis of consecutive ED visits, implementation of the ED-ICU was associated with reduced 30-day mortality (aOR 0.85, 95% CI 0.80–0.90).^6^ Further, in a matched cohort from the same institution, the opening of an ED-ICU was associated with lower 60-day mortality (hazard ratio 0.84, 95% CI 0.70–0.99).^25^ When focusing on patients with acute stroke, the number of patients with favorable clinical outcomes increased after the opening of an ED-ICU.^14^

Conversely, several other studies highlighted that creating an ED-ICU did not necessarily translate to improved survival.^8^ ^,^ ^12^ ^,^ ^26^ ^,^ ^30^ When analyzed using different subgroups (ie, subgroups of patients with upper GI bleeding or decompensating patients), the data from the ED-ICU that previously exhibited survival benefit did not show differences in mortality between the pre- or post-implementation cohorts.^26^ ^,^ ^30^ Among patients with acute respiratory failure or sepsis, the implementation of an ED-ICU was not associated with in-hospital survival.^8^ Finally, a study showed increased mortality among patients admitted to the ED-ICU compared with patients admitted to other inpatient ICUs. This could be due to a higher proportion of patients with a higher burden of comorbid conditions or higher severity of disease admitted to the ED-ICU (compared to the inpatient ICU).^12^

### Cost-Related Outcomes

One study explored the association of ED-ICU and cost-related outcomes. Establishing the ED-ICU was associated with increased inflation-adjusted net revenue per encounter: 7% (95% CI, 3.5–10.6%) and without increased inflation-adjusted cost.^29^

## DISCUSSION

In this scoping review, we identified that several ED-ICUs have been established around the world with both unique and shared characteristics. Overall, these ED-ICUs are characterized by the provision of early and continued ICU-level care and are generally staffed by emergency physicians, nurses, and interdisciplinary team members. Implementing an ED-ICU model may be associated with timely ICU-level care, as well as reductions in inpatient ICU admission rates without compromising clinical patient outcomes.

Importantly, despite sharing several characteristics, both LOS and mortality risk varied greatly across reported ED-ICUs. This variability could be due to different utilization of ED-ICUs and different case mix (eg, higher LOS associated with the care of patients who have a higher degree of severity at baseline, while shorter LOS may be associated with lower overall severity or transitioning to palliative care). Notably, we identified ED-ICUs as being predominantly a 1) short-stay ICU; 2) a palliative-care ICU; or a 3) disaster-response ICU. The latter type was fueled by lack of appropriate resources in the time of a disaster, whereas the short-stay and palliative-care ICUs were mostly initiated due to a lack of inpatient ICU beds. As short-stay ICU admissions generally comprise a high proportion of all ICU admissions, diverting these patients to an ED-ICU model could preserve important inpatient ICU capacity.^36^ The potential diagnoses that could be served under a short-stay ED-ICU admission model may include acute poisoning, intoxication, upper GI bleeding, DKA, minor strokes, and transient cerebral ischemia.^17^ ^,^ ^18^ ^,^ ^22^ ^,^ ^23^ ^,^ ^26^ ^,^ ^36^ ^,^ ^37^ On the other end of the spectrum, patients who are unlikely to benefit from a prolonged trial of invasive, life-sustaining interventions could also be assessed and cared for under an ED-ICU model that can then serve as a transition to a different pathway such as a palliative-care approach. Such implementations could potentially explain the high mortality rate observed in some of these ED-ICUs where terminally ill patients are admitted.^10^ ^,^ ^12^

In this review, we identified that establishing an ED-ICU was potentially associated with improvement in several process measures, including time to ICU-level care, reduction of inpatient ICU admission rates, and increased delivery of evidence-based treatments. However, whether this translates to clinically relevant patient-related outcomes remains unclear given that 1) studies report conflicting data on patient-related outcomes and 2) the high likelihood of confounding by indication.^38^ For example, the higher mortality rate observed within some ED-ICUs might be due to confounding due to baseline severity; patients with higher burden of comorbid conditions and severity at baseline (who are at higher risk of in-hospital death) might be selectively admitted to the ED-ICUs.^10^ ^,^ ^12^ Moreover, whether the implementation of an ED-ICU model can be expected to impact clinical outcomes beyond process measures (compared to timely ICU-level care delivery elsewhere) remains unclear.^39^

Although establishing an ED-ICU model may lead to improved timely ICU-level care and preservation of inpatient ICU capacity, adopting an ED-ICU model requires multiple considerations. First, institutional needs assessment should be performed by focusing on identifying key stakeholders, evaluating ED throughput, and determining bed capacity and availability of resources.^5^ Second, the financing and staffing model should not be neglected. Finally, healthcare leaders should also consider alternative solutions to an ED-ICU model, including improved institutional policy, personnel-focused intervention, or increased number of inpatient ICU beds.

## LIMITATIONS

Several limitations need to be considered when evaluating our findings. First, it is likely that many more ED-ICUs exist than those reported in the literature. For example, there were at least three more ED-ICUs in the US than was reported in the indexed literature.^40^ It remains unknown whether the reported ED-ICU models represent the latter, unreported group. Additionally, we did not include a commonly cited ED-ICU implementation because it did not meet our definition of an ED-ICU model, as it was located farther away from the ED and specifically designed to accept critical care transfers from other hospitals.^41^ ^,^ ^42^ Second, most of the reported outcomes associated with ED-ICU operation were from one site (with 18 related studies) of the identified 31 papers. This could potentially limit the generalizability of our findings. Third, the reported outcomes were largely obtained from retrospective cohorts, with several limitations in their design; most studies did not provide sample-size determination, and were subject to unmeasured and residual confounding as well as outcome misclassification. Fourth, ideally, the data from each study should be pooled and analyzed collectively. However, due to the significant heterogeneity in data reporting, patient inclusion, and severity of disease across studies, summary statistics were not calculated for all reported data.

## CONCLUSION

Several ED-ICUs have been established around the world with different characteristics depending on local needs. Implementation of the ED-ICU may be associated with improved process measures; however, its impact on clinical outcomes remains not fully characterized. Importantly, future research should focus on how the establishment of an ED-ICU could be useful in different contexts and geographical areas, alongside its related success factors. Furthermore, cost-related outcomes should be further explored, as they are important considerations for stakeholders in adopting an ED-ICU model.

## Supplementary Information




