# Supplementary material for: Characteristics and Outcomes of Implementing Emergency Department-based Intensive Care Units: A Scoping Review
Source: West J Emerg Med. 2024 Nov 27;26(1):78–85. doi: 10.5811/westjem.24874 (PMC11908510; doi:10.5811/westjem.24874)
Supplement: Supplementary file 1 [file wjem-26-78-s001.docx]

**Supplementary appendix**

**Table S1** Search strategy

**Ovid MEDLINE: Epub Ahead of Print, In-Process & Other Non-Indexed Citations, Ovid MEDLINE® Daily and Ovid MEDLINE® <1946-Present>**

| # | Searches | Comment |
| --- | --- | --- |
| 1 | emergency medicine/ or pediatric emergency medicine/ |  |
| 2 | emergency service, hospital/ or trauma centers/ |  |
| 3 | (emergency adj2 (department* or room* or service* or ward* or unit*)).ti,ab,kf. |  |
| 4 | 1 or 2 or 3 | ED department terms |
| 5 | intensive care units/ or respiratory care units/ |  |
| 6 | ((intensive or critical or resuscitat*) adj2 (unit* or department* or ward* or room* or service*)).ti,ab,kf. |  |
| 7 | 5 or 6 | ICU terms |
| 8 | "length of stay"/ or patient admission/ or patient discharge/ or patient readmission/ or patient transfer/ |  |
| 9 | ("length of stay" or (patient adj2 (admission or discharge or readmission or transfer))).ti,ab,kf. |  |
| 10 | 8 or 9 | length of stay or admission terms |
| 11 | 4 and 7 and 10 | base set 1 |
| 12 | (emergency adj2 department adj2 based adj2 (intensive or icu)).ti,ab,kf. |  |
| 13 | ed icu.ti,ab,kf. |  |
| 14 | (intensive adj2 care adj2 team*).ti,ab,kf. |  |
| 15 | 4 and 14 |  |
| 16 | 12 or 13 or 15 | ed based icu results |
| 17 | 11 or 16 |  |

**Table S2** Characteristics of Emergency department-based intensive care units (ED-ICUs)

| **Name** | **Country** | **Institution** | **Number of publications** | **Sources of patients** | **Patient population** | **Reason for initiation** | **Admission criteria** | **ICU Beds** | **Staffing** |
| --- | --- | --- | --- | --- | --- | --- | --- | --- | --- |
| Emergency Critical Care Center (EC3) | United States | University of Michigan | 12 retrospective cohorts^6,18-22,25-30^  , 1 systematic review^31^,  4 reviews^2,5,32,33^  , 1 commentary^35^ | Outpatient | General | ED Boarding of ICU patients | Ongoing critical care need (even when other ICU bed is available) | 9 | 1 EM attending physician (with or without CCM board),  1 or 2 residents, fellows, or physician assistants,  The patient-to-nurse ratio is 1:1 or 2:1, presence of RTs and pharmacists (shared with ED) |
| Resuscitation and Critical Care Unit (ResCCU) | United States | Hospital of the University of Pennsylvania | 3 retrospective cohorts ^8,17,23^  , 3 reviews^2,5,32^ | Outpatient | General | ED Boarding of ICU patients | Ongoing critical care need  (even when other ICU bed is available) | 6 | EM physicians, EM-trained intensivists, EM house staff, ED or ICU-trained nurses, respiratory therapists, and clinical pharmacists  (staffed only on weekdays: Monday 7:00 A.M. to Saturday 7:00 A.M) |
| Resuscitation and Acute Critical Care Unit (RACC) | United States | Stony Brook University Medical Center | 4 reviews^2,5,32,33^ | Outpatient | General | ED Boarding of ICU patients | Ongoing critical care (when an ICU bed is not available) | 3 | EM physicians. 2-3 EM residents for 19 hr daily then 1 resident for 5 hr, 2  resuscitation fellows, maximum nurse-to-patient ratio is 1:2 |
| ED intensive care unit (EDICU) | Turkey | Hacettepe University | 1 prospective cohort^10^, 1 systematic review^31^ | Outpatient | General | ED Boarding of ICU patients | Ongoing critical care (when an ICU bed is not available) | 8 | An EM specialist, a senior resident, and 2 nurses |
| ED ICU | France | Amiens University Medical Center | 1 retrospective cohort^14^, 1 review^5^ | Outpatient | General, especially stroke | To manage general critical care esp. stroke patients |  | 6 | EM physicians, nurses, nursing assistants |
| Emergency department intensive care unit (EDICU) | Taiwan | Chang Gung Memorial Hospital | 1 retrospective cohort^12^, 1 systematic review^31^ | outpatient | General | ED Boarding of ICU patients | Ongoing critical care (when an ICU bed is not available) | 14 | EPs with CCM board |
| Shock room | Belgium | Erasme University Hospital | 1 retrospective cohort^11^ | Inpatient and Outpatient | General | facilitating rapid diagnosis and management of all acutely ill patients | Unstable patients | 4 | A senior ED physician or an intensivist,  two nurses |
| ED-ICU | Brazil | Instituto Central do Hospital das Clínicas da Faculdade de Medicina da Universidade de São Paulo (IC-HCFMUSP) | 1 retrospective cohort^15^ | outpatients | General | - | Ongoing critical care (when an ICU bed is not available) | 17 | Not reported |
| Emergency ICU (EICU) | Republic of Korea | Seoul National University Hospital, | 1 retrospective cohort^7^ | outpatients | General | ED Boarding of ICU patients | Ongoing critical care need  (even when other ICU bed is available) | 12 | Five EP (3 certified, 2 in training for CCM), 2 EM residents.  The ratio of nurses to patients was 1:2 |
| Emergency ICU (EICU) | Taiwan | Taipei Veterans General Hospital | 1 retrospective cohort^24^ | outpatient | General | ED Boarding of ICU patients | Ongoing critical care (when an ICU bed is not available) | 13 | Not reported |
| ED-ICU | Taiwan | Taipei Medical University Hospital | 1 retrospective cohort^16^ | Inpatient and Outpatient | General | Reservoir of critical care for hospital | Ongoing critical care | 8 | Physicians trained in EM and CCM |
| Emergency Intensive Care Unit (EICU) | United States | Bellevue Hospital Center | 1 retrospective cohort^13^ | Outpatient | General | Disaster (Hurricane) | Ongoing critical care before transfer | 10 | EM physician trained in CCM, 1 PGY-4, 2 PGY-1 EM residents, a nurse manager, RTs, critical care pharmacy and pharmacist services |
| ED-ICU | United States | Mount Sinai Hospital | 1 report^34^ | Outpatient | General, esp.COVID-19 | Disaster (COVID-19) | Ongoing critical care need | 13 | EM attending and two either PGY-2 or above EM residents or senior physician assistants,  5 nurses for 13 ICU beds and 14 step-down beds with additional 3 nurses 12 hours per day,  two RTs with a third added for 12 hours a day,  3 ED technicians |
| Not reported | United States | Henry Ford Hospital | 1 review^5^ | Outpatient | General | ED Boarding of ICU patients | Ongoing critical care (when an ICU bed is not available) | 16 | A senior EP attending, a PGY-3 or above EM or IM resident, a PGY-2 resident, nurses, an RT, a pharmacist |

Abbreviations: ED: emergency department; EM: emergency medicine; EP: emergency physician; CCM: critical care medicine; COVID-19: coronavirus disease 2019; ICU: intensive care unit; IM: internal medicine; PGY: post-graduate year; RT: respiratory therapist

**Table S3** Details of paper included in this scoping review.

| **Study author and year** | **Country** | **Institution of the ED-ICU** | **Study design** | **Aim of the paper** |
| --- | --- | --- | --- | --- |
| Aslaner et al. 2015^10^ | Turkey | Hacettepe University | Prospective cohort | To determine which type of critically ill patients play a main role for crowding in the ED-ICU, and how to manage these patients. |
| Piagnerelli et al. 2009^11^ | Belgium | Erasme University Hospital | Retrospective cohort | To document experiences in the shock room over a one-year period. |
| Tseng et al. 2015^12^ | Taiwan | Chang Gung Memorial Hospital | Retrospective cohort | To report comparative outcomes of ED-ICU patients with specialty ICU patients. |
| Smith et al. 2016^13^ | United States | Bellevue Hospital Center | Retrospective cohort | To assess the impact of a contingency emergency intensive care space established as part of a freestanding emergency department during the aftermath of Hurricane Sandy. |
| Puy et al. 2017^14^ | France | Amiens University Medical Center | Retrospective cohort | To assess the effects of organizational and logistic changes on acute stroke management following the  creation of an ICU inside conventional adult Emergency Department (ED-ICU) of a university medical center |
| Correa da Costa Ribeiro et al. 2018^15^ | Brazil | Instituto Central do Hospital das Clínicas da Faculdade de Medicina da Universidade de São Paulo (IC-HCFMUSP) | Retrospective cohort | To assess the criterion validity and inter-rater reliability of a palliative care screening tool for patients admitted to an ED-ICU. |
| Wang et al. 2019^16^ | Taiwan | Taipei Medical University | Retrospective cohort | To examine the characteristics and outcomes of critical illness in patients with prior DNR who were admitted to the ED-ICU in an Asian society. |
| Gunnerson et al. 2019^6^ | United States | University of Michigan | Retrospective cohort | To determine the association of a novel ED-based ICU with 30-day mortality and inpatient ICU admission. |
| Zhou et al. 2019^17^ | United States | Hospital of the University of Pennsylvania | Retrospective cohort | To identify which patients presenting with DKA required short-term ICU care of <24 h and who therefore are ideal for treatment in the ED-ICU setting. |
| Jeong et al. 2020^7^ | Republic of Korea | Seoul National University Hospital | Retrospective cohort | To evaluate the efficacy of our emergency physician intensivists-based ED-ICU system. |
| Haas et al. 2020^18^ | United States | University of Michigan | Retrospective cohort | To evaluate the impact of an ED-ICU on disposition outcomes for adult ED patients in DKA. |
| Haas et al. 2020^19^ | United States | University of Michigan | Retrospective cohort | To descriptively analyze characteristics and outcomes of patients extubated in an ED-ICU. |
| Haas et al. 2020^20^ | United States | University of Michigan | Retrospective cohort | To examine patterns of consultation to the ED-ICU and their relationship with shift turnover times in the main ED. |
| Leith et al. 2020^21^ | United States | University of Michigan | Retrospective cohort | To descriptively analyze patients receiving end-of-life care in an ED-ICU. |
| Joseph et al. 2020^22^ | United States | University of Michigan | Retrospective cohort | To present a descriptive analysis of experience of implementing a novel protocol using ED-ICU for management of minor intracranial hemorrhage patients in the ED setting. |
| Anesi et al. 2020^8^ | United States | Hospital of the University of Pennsylvania | Retrospective cohort | To evaluate the potential impact of ED-ICU among patients with sepsis and acute respiratory failure. |
| Mudan et al. 2020^23^ | United States | Hospital of the University of Pennsylvania | Retrospective cohort | To identify characteristics of poisoned patients treated in the ED-ICU. |
| Chang et al. 2021^24^ | Taiwan | Taipei Veterans General Hospital | Retrospective cohort | To determine if the early integration of palliative care in the ED resulted in a difference in the hospital care, LOS, and mortality between the patients receiving palliative care and those receiving usual care. |
| Du et al. 2021^25^ | United States | University of Michigan | Retrospective cohort | To examine how the establishment of the ED-ICU influenced the number and type of admissions to the critical care medicine unit and their outcomes. |
| Haas et al. 2021^26^ | United States | University of Michigan | Retrospective cohort | To determine how an ED-ICU impacts patient and resource utilization outcomes for critically ill  ED patients with upper gastrointestinal bleeding. |
| Harvey et al. 2022^27^ | United States | University of Michigan | Retrospective cohort | To determine rates of adherence to a multicomponent lung protective ventilation strategy for adult ED patients undergoing mechanical ventilation before and after implementation of an ED-ICU. |
| Puls et al. 2022^28^ | United States | University of Michigan | Retrospective cohort | To analyze the impact of ED length of stay before transfer to an ED-ICU on patient outcomes. |
| Bassin et al. 2022^29^ | United States | University of Michigan | Retrospective cohort | To determine the association of an ED-ICU with inflation-adjusted change in mean direct cost of care, net revenue, and direct margin per ED patient encounter. |
| Doan et al. 2023^30^ | United States | University of Michigan | Retrospective cohort | To analyze the outcomes of patients who decompensate while boarding in the ED requiring escalation to ICU-level care, comparing patients managed in the ED-ICU to those who were not. |
| McDowald et al. 2017^31^ | United States | Hacettepe University, Chang Gung Memorial Hospital, University of Michigan | Systematic review | To evaluate the effectiveness of collaboration between the ED and ICUs on the mortality rates of critically ill adult ED patients. |
| Leibner et al. 2019^32^ | United States | University of Michigan , Stony Brook University Medical Center, Hospital of the University of Pennsylvania | Narrative review | To describe and contrast the mission, staffing, patient selection, and services provided by resuscitative care units. |
| Jayaprakash et al. 2020^5^ | United States | University of Michigan , Stony Brook University Medical Center, Hospital of the University of Pennsylvania, Henry Ford Hospital, Amiens University Medical Center | Narrative review | To provide a narrative review of different models of delivery of critical care reported in the literature and  highlight aspects for consideration for successful local implementation. |
| Mohr et al. 2020^2^ | United States | University of Michigan , Stony Brook University Medical Center, Hospital of the University of Pennsylvania | Narrative review | To review the United States literature on the frequency of emergency department boarding among the critically ill, the outcomes associated with critical care patient boarding, and local strategies developed  to mitigate the impact of emergency department critical care boarding on patient outcomes. |
| Mermiri et al. 2021^33^ | Greece | University of Michigan , Stony Brook University Medical Center | Narrative review | To narrate critical emergency medicine and the resuscitative care unit. |
| Hickey et al. 2020^34^ | United States | Mount Sinai Hospital | Report | To detail the ED’s rapid structural changes, staffing transition, and physical updates for critically ill patients to offer lessons learned during COVID-19 pandemic. |
| Kurz et al. 2019^35^ | United States | University of Michigan | Commentary | To comment on the work of Gunnerson et al.^6^ |

Abbreviations: ED: emergency department; ED-ICU: emergency department-based intensive care unit; COVID-19: coronavirus disease 2019; DKA: diabetic ketoacidosis; DNR: Do-Not-Resuscitate; ICU: intensive care unit
